# Comparing hemodynamic models with DCM

**DOI:** 10.1016/j.neuroimage.2007.07.040

**Published:** 2007-11-15

**Authors:** Klaas Enno Stephan, Nikolaus Weiskopf, Peter M. Drysdale, Peter A. Robinson, Karl J. Friston

**Affiliations:** a Wellcome Trust Centre for Neuroimaging, Institute of Neurology, University College London, 12 Queen Square, London WC1N 3BG, UK; b School of Physics, University of Sydney, NSW 2006, Australia; c Brain Dynamics Center, Westmead Millennium Institute, Westmead Hospital and Western Clinical School of the University of Sydney, Westmead, NSW 2145, Australia; d Faculty of Medicine, University of Sydney, NSW 2006, Australia

**Keywords:** Dynamic causal modeling, Balloon model, Effective connectivity, BOLD signal, Bayesian model selection, System identification

## Abstract

The classical model of blood oxygen level-dependent (BOLD) responses by Buxton et al. [Buxton, R.B., Wong, E.C., Frank, L.R., 1998. Dynamics of blood flow and oxygenation changes during brain activation: the Balloon model. Magn. Reson. Med. 39, 855–864] has been very important in providing a biophysically plausible framework for explaining different aspects of hemodynamic responses. It also plays an important role in the hemodynamic forward model for dynamic causal modeling (DCM) of fMRI data. A recent study by Obata et al. [Obata, T., Liu, T.T., Miller, K.L., Luh, W.M., Wong, E.C., Frank, L.R., Buxton, R.B., 2004. Discrepancies between BOLD and flow dynamics in primary and supplementary motor areas: application of the Balloon model to the interpretation of BOLD transients. NeuroImage 21, 144–153] linearized the BOLD signal equation and suggested a revised form for the model coefficients. In this paper, we show that the classical and revised models are special cases of a generalized model. The BOLD signal equation of this generalized model can be reduced to that of the classical Buxton model by simplifying the coefficients or can be linearized to give the Obata model. Given the importance of hemodynamic models for investigating BOLD responses and analyses of effective connectivity with DCM, the question arises which formulation is the best model for empirically measured BOLD responses. In this article, we address this question by embedding different variants of the BOLD signal equation in a well-established DCM of functional interactions among visual areas. This allows us to compare the ensuing models using Bayesian model selection. Our model comparison approach had a factorial structure, comparing eight different hemodynamic models based on (i) classical vs. revised forms for the coefficients, (ii) linear vs. non-linear output equations, and (iii) fixed vs. free parameters, ε, for region-specific ratios of intra- and extravascular signals. Using fMRI data from a group of twelve subjects, we demonstrate that the best model is a non-linear model with a revised form for the coefficients, in which ε is treated as a free parameter.

## Introduction

In many models of effective connectivity, like structural equation modeling (Horwitz et al., 1999; McIntosh and Gonzalez-Lima, 1994; Büchel and Friston, 1997), psychophysiological interactions (Friston et al., 2007), or multivariate autoregression (Harrison et al., 2003; Goebel et al., 2003), coupling parameters are estimated directly from the measured time-series. However, the causal system architecture one wishes to identify exists at the level of neuronal dynamics. In modeling dependencies among measured data, one implicitly assumes that neural activity can be observed directly. This is tenable for single neuron recordings, but not for non-invasive techniques like functional magnetic resonance imaging (fMRI) or electroencephalography (EEG). For example, in fMRI, the relationship between neural activity and measured blood oxygen level dependent (BOLD) signals is complicated and not fully understood (Attwell and Iadecola, 2002; Lauritzen, 2004; Logothetis et al., 2001; Logothetis and Wandell, 2004). In short, the interpretation of models of effective connectivity, which ignore the transformation from the neural activity to the BOLD measurements, can be difficult (Gitelman et al., 2003).

**Fig. 1:**
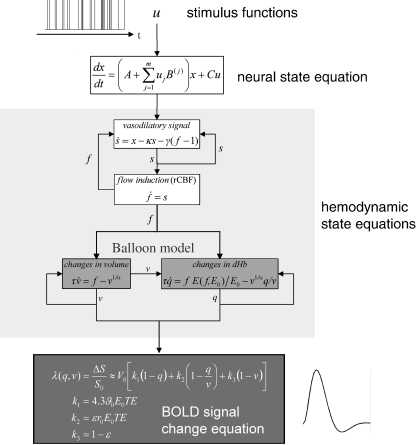
Schematic summary of the hemodynamic forward model in DCM. Experimentally controlled input functions *u* evoke neural responses *x*, modeled by a bilinear differential state equation, which trigger a hemodynamic cascade, modeled by 4 state equations with 5 parameters. These hemodynamic parameters comprise the rate constant of the vasodilatory signal decay (*κ*), the rate constant for autoregulatory feedback by blood flow (*γ*), transit time (*τ*), Grubb's vessel stiffness exponent (*α*), and capillary resting net oxygen extraction (*ρ*). The so-called Balloon model consists of the two equations describing the dynamics of blood volume (*ν*) and deoxyhemoglobin content (*q*) (light grey boxes). Integrating the state equations for a given set of inputs and parameters produces predicted time-series for ν and *q* which enter a BOLD signal equation *λ* (dark grey box) to give a predicted BOLD response. Here, we show the equations of the RBM_N_(*ε*) model from this paper. For parameter estimation, an observation model is used that treats the observed BOLD response as a function of inputs and parameters plus some observation error (see main text).

To enable valid inferences about connectivity at the neural level, effective connectivity models have to combine a model of neural dynamics with a biophysically plausible forward model that describes the transformation from neural activity to measured signals. The inversion of such models furnishes estimates of both neural and forward model parameters (see Stephan et al., 2004 for review). Dynamic causal modeling (DCM) of fMRI data uses a model that conforms to this principle (Friston et al., 2003).^1^ DCM uses a hemodynamic forward model that is based on work by Buxton et al. (1998) and its extension by Friston et al. (2000). This model consists of three parts (Fig. 1 ). The first describes the link between neural activity and regional cerebral blood flow (rCBF) and is based on linear differential equations modeling a dampened oscillator: changes in neural activity elicit an exponentially decaying vasodilatory signal that is subject to feedback-regulation by the flow it induces (Friston et al., 2000). The second part concerns the dependence of the BOLD signal on rCBF-induced changes of blood volume and deoxyhemoglobin content. This so-called “Balloon model” (Buxton et al., 1998) describes the behavior of the post-capillary venous compartment by analogy to an inflated Balloon, postulating a non-linear dependence of BOLD signal on blood volume *ν* and deoxyhemoglobin content *q.* Together, these two components represent the hemodynamic state equations which have been the focus of many empirical and theoretical investigations of the BOLD response (e.g., Deneux and Faugeras, 2006; Riera et al., 2004, 2006; Robinson et al., 2006) and, as a core component of DCM, find widespread use in analyses of effective connectivity (e.g., Bitan et al., 2005; Mechelli et al., 2004; Lee et al., 2006; Stephan et al., 2007a). Finally, the output or BOLD signal change equation *λ*(*q*,*ν*) links *ν* and *q* to BOLD signal change (Fig. 1). This paper is only concerned with the last part of the overall hemodynamic forward model; i.e., different variants of the BOLD signal change equation. For brevity, we will refer to the BOLD signal change equation as the “BOLD model” (BM) throughout the paper.

Over the last years, a variety of extensions (and alternatives) have been proposed for both the hemodynamic state equations and the BM (e.g., Aubert and Costalat, 2002; Davis et al., 1998; Mandeville et al., 1999; Sotero and Trujillo-Barreto, 2007; Uludag et al., 2004; Zheng et al., 2002). In particular, Buxton et al. (2004) and Obata et al. (2004) suggested a revision of their original formulation of the BM. Compared to the classical BM (Buxton et al., 1998), Obata et al. (2004) assumed a linear dependence of BOLD signal on blood volume *v* and deoxyhemoglobin content *q*. Furthermore, they offered revised definitions of the coefficients, which were based on more recent experimental data and depended explicitly on acquisition-specific parameters like echo time (TE).

Given the importance of hemodynamic modeling for investigations of BOLD responses and analyses of effective connectivity with DCM, the question arises: which formulation is a more appropriate model of empirically measured BOLD responses? In this article, we address this question by embedding different variants of the BM in a well-established DCM of functional interactions in the ventral stream of the visual cortex (Stephan et al., 2007a), which is fitted to BOLD responses that were measured in a group of twelve subjects (Stephan et al., 2003). We then compare the DCMs with different BMs by means of Bayesian model selection (BMS; Penny et al., 2004a). Specifically, we addressed the following questions:

- Is the more recent model by Obata et al. (2004) a better model than the classical model by Buxton et al. (1998) for measured BOLD responses?
- Can the models be improved further by treating *ε*, the ratio of intra- and extravascular signal, as a free parameter that is estimated from the data?
- Can a non-linear variant of the revised model be derived by requiring additional consistency in the approximations and does this variant outperform other models?

Using fMRI data from a group of 12 subjects (Stephan et al., 2003), we demonstrate that the best model is a non-linear model with a revised form for the coefficients, in which *ε* is treated as a free parameter. In what follows, we review the hemodynamic model, dynamic causal modeling and Bayesian model selection. We then consider the BM variants that are evaluated in the final section.

## Theory

In the treatment of the hemodynamic state variables below, we follow Buxton et al. (1998) and Friston et al. (2000) in using capital letters for specific dimensional values of a state variable and lower case letters for normalized state variables (normalized to their values at rest).

### The classical BOLD signal model by Buxton et al. (1998)

In their pioneering work, Buxton et al. (1998) modeled the BOLD signal at rest, *S*_0_, as a sum of intravascular (*S*_I_) and extravascular (*S*_E_) signal, weighted by resting venous blood volume fraction *V*_0_:

(1) $$S_{0}=(1-V_{0})S_{\text{E}}+V_{0}S_{\text{I}}$$

Based on theoretical and empirical results, Buxton et al. (1998) derived the following non-linear equation for BOLD signal change Δ*S* during activation, relative to resting signal *S*_0_ (see lower part of Fig. 1):

(2) $$\lambda (q\text{,}v)=\frac{\Delta S}{S_{0}}\approx V_{0}\left[k_{1}(1-q)+k_{2}\left(1-\frac{q}{v}\right)+k_{3}(1-v)\right]\text{,}$$

$$k_{1}=7E_{0}\text{,}$$

$$k_{2}=2\text{,}$$

$$k_{3}=1-\varepsilon \approx 2E_{0}-0.2$$

In this BM, *ν* and *q* are the venous blood volume and deoxyhemoglobin content (both normalized to their values at rest), and *E*_0_ is the oxygen extraction fraction at rest. The coefficients *k*_1_...*k*_3_ were estimated assuming a field strength of 1.5 T and a TE of 40 ms. At first glance, the BOLD equation in Buxton et al. (1998) does not seem to possess anything analogous to *ε*, the ratio of intra- and extravascular signal. However, the derivation of this equation in the appendix to Buxton et al. (1998) includes a parameter *β* which is equivalent to ε, but is substituted in their final equation by an approximation based on results by Boxerman et al. (1995). Without this approximation, one obtains *k*_3_ = 1 − *ε*. Buxton et al. (1998) used fixed values *E*_0_ = 0.4 and *ε* = 0.4 for which *k*_3_ = 2*E*_0_ − 0.2 = 1 − *ε.*

The coefficients *k*_1_...*k*_3_ in Eq. (2), as published in Buxton et al. (1998) and used subsequently in many studies, can be improved in two ways. The first improvement is to drop any assumptions about TE and use the full expressions that Buxton and colleagues derived initially and then simplified. The second improvement involves correcting an error in the derivation of the coefficient *k*_2_. This error was mentioned in the later article by Obata et al. (2004) but its nature was not specified. Additionally, we use a more accurate expression for *k*_1_ that does not rely on the assumption that *V*_0_ is very small. Together, these improvements lead to the following expressions for the coefficients:

(3) $$k_{1}=(1-V_{0})4.3\vartheta_{0}E_{0}TE\text{,}$$

$$k_{2}=2E_{0}\text{,}$$

$$k_{3}=1-\varepsilon$$

Here, *ϑ*_0_ = 40.3 s^−1^ is the frequency offset at the outer surface of the magnetized vessel for fully deoxygenated blood at 1.5 T. In this paper, any results for models based on the classical formulation by Buxton et al. (1998) use this improved form of the coefficients as shown in Eq. 3.^2^

#### The balloon model

The BM described above requires that one knows the evolution of blood volume *ν* and deoxyhemoglobin content *q*. The Balloon model by Buxton et al. (1998) proposed differential equations for *ν* and *q*, based on two main assumptions about the biophysical mechanisms involved: (i) small post-capillary vessels react to an increase in inflowing blood like an inflating balloon, and (ii) oxygen extraction is tightly coupled to blood flow. The first assumption means that changes in (normalized) venous blood volume *ν* correspond to differences in inflow *f*_in_ and outflow *f*_out_ with a time constant *τ*:

(4) $$\tau \frac{dv}{dt}=f_{\text{in}}(t)-f_{\text{out}}(v)$$

Here, *τ* is the mean transit time of blood, i.e., the average time blood takes to traverse the venous compartment, and corresponds to the ratio of resting blood volume *V*_0_ to resting blood flow *F*_0_: *τ* = *V*_0_/*F*_0_. Following the results by Grubb et al. (1974) 3 , outflow is modeled as a function of volume with a single parameter *α* that represents the resistance of the venous balloon, i.e., vessel stiffness:

(5) $$f_{\text{out}}(v)=v^{1/\alpha }$$

The second assumption above (i.e., that oxygen extraction is tightly coupled to blood flow) determines the equation for deoxyhemoglobin content *q*. Generally, the change in *q* corresponds to the delivery of deoxyhemoglobin into the venous compartment, minus that expelled. Assuming fully oxygenated hemoglobin in pre-capillary blood, the delivery of deoxyhemoglobin into the venous compartment corresponds to the product of blood inflow and the oxygen extraction fraction *E*, whereas its clearance is the product of outflow and deoxyhemoglobin concentration *q*/*ν*:

(6) $$\tau \frac{dq}{dt}=f_{\text{in}}(t)\frac{E(f_{\text{in}}\text{,}E_{0})}{E_{0}}-f_{\text{out}}(v)\frac{q(t)}{v(t)}$$

Assuming that any extracted oxygen is metabolized immediately, thereby maintaining a tissue oxygen concentration of zero, the oxygen gradient across the capillary wall and oxygen extraction rate depends entirely on oxygen delivery, and thus on blood flow. Buxton and Frank (1997) showed that a reasonable approximation across a wide range of conditions is

(7) $$E(f_{\text{in}})=1-{\left(1-E_{0}\right)}^{1/f_{\text{in}}}$$

Together, these considerations led Buxton et al. (1998) to propose the following state equations for *v* and *q* (from now on, blood inflow will simply be denoted as *f*):

(8) $$\tau \frac{dv}{dt}=f(t)-v{\left(t\right)}^{1/\alpha }$$

$$\tau \frac{dq}{dt}=f(t)\frac{1-{\left(1-E_{0}\right)}^{1/f}}{E_{0}}-v{\left(t\right)}^{1/\alpha }\frac{q(t)}{v(t)}$$

#### Neurovascular state equations

Eq. (8) shows that the only state variable that *ν* and *q*, and thus the BOLD signal, depend on is blood inflow *f*. The issue of how blood flow depends on neural activity was addressed by Friston et al. (2000). In their completion of the Buxton model, vascular responses to neural activity correspond to a dampened oscillator: changes in neural activity *x* elicit an exponentially decaying vasodilatory signal s that is also subject to feedback regulation by the flow f it induces (Fig. 1):

(9) $$\frac{ds}{dt}=x-\kappa s-\gamma (f-1)$$

(10) $$\frac{df}{dt}=s$$

Here, *κ* and *γ* are the rate constants of signal decay and feedback regulation, respectively. Note that *f* is normalized flow with regard to resting flow *F*_0_, and therefore the feedback regulation term (*f* − 1) in Eq. (9) becomes zero during rest.

This linear model of the relation between neural activity and rCBF concurs with several experimental results (Friston et al. 1998; Miller et al. 2001). In particular, the results of combined perfusion and BOLD measurements by Miller et al. (2001) during various stimulation conditions were “consistent with a model with a non-linear step from stimulus to neural activity, a linear step from neural activity to CBF change, and a non-linear step from CBF change to BOLD signal change.” This is exactly what the hemodynamic model represents. Note that neural activity x, driving the vasodilatory signal in Eq. (9), is the output from the neural state equation in DCM (see Fig. 1 and below). This provides for a flexible model of the link between neural activity and rCBF that can capture a large variety of transients, sustained responses, and adaptation effects—in brief, any kind of neural dynamics that can be modeled with bilinear differential equations as used in DCM (see Fig. 1 in Penny et al. 2004b and Fig. 5 in Stephan et al. 2007b for examples).

### Revised coefficients for the BOLD signal model

Buxton and colleagues (Obata et al. 2004) recently proposed a revised form of the classical BM (Eq. (2)). The critical changes they proposed were (i) a linear dependence of BOLD signal on blood volume *v* and deoxyhemoglobin content *q* and (ii) new coefficients *k*_1_...*k*_3_ which were computed on the basis of more recent experimental data and which explicitly consider acquisition-specific TE and the ratio of intra- and extravascular signals (*ε*):

(11) $$\frac{\Delta S}{S_{0}}\approx V_{0}[(k_{1}+k_{2})(1-q)+(k_{2}+k_{3})(1-v)]\text{,}$$

$$k_{1}=4.3\vartheta_{0}E_{0}TE\text{,}$$

$$k_{2}=\varepsilon r_{0}E_{0}TE\text{,}$$

$$k_{3}=\varepsilon -1$$

According to Obata et al. (2004), for a field strength of 1.5 T, *r*_0_ = 25 s^−^^1^ is the slope of the relation between the intravascular relaxation rate *R*_2I_* and oxygen saturation. Furthermore, they chose fixed values for the resting oxygen extraction fraction (*E*_0_ = 0.4) and for the ratio of intra- and extravascular signal (*ε* = 1.43).

### A generalized BOLD signal model

Both the classical BM by Buxton et al. (1998) and the more recent version by Obata et al. (2004) can be regarded as special cases of the following generalized BM:

(12) $$\frac{\Delta S}{S_{0}}\approx V_{0}\left[k_{1}(1-q)+k_{2}\left(1-\frac{q}{v}\right)+k_{3}(1-v)\right]$$

$$k_{1}=4.3\vartheta_{0}E_{0}TE$$

$$k_{2}=\varepsilon r_{0}E_{0}TE$$

$$k_{3}=1-\varepsilon$$

This model is derived in Appendix B, starting from the same point as Obata et al. (2004), but using more consistent approximations. It retains the non-linear output equation in the classical Buxton model and combines it with the revised coefficients from the Obata model. It is more general than these two models: its coefficients can be reduced to those of the classical model, and its derivation can be simplified to yield the Obata model (see Appendix B). This model is a useful starting point to consider a number of BM variants that differ in terms of (i) classical (Buxton) vs. revised (Obata) forms for the coefficients, (ii) linear vs. non-linear output equations, and (iii) fixed vs. free parameters, *ε*, for the ratio of intra- and extravascular signals. These variants, all of which we fitted to empirical data and compared using BMS, are described in the next three sections. The nomenclature of these models is summarized in Table 1 .

#### Classical vs. revised forms for the coefficients

The classical model obtains by simplifying the revised expressions for the coefficients in Eq. (12) through reduction: it assumes a fixed TE of 40 ms and omits the dependence of *k*_2_ on *ε*, the ratio of intra- and extravascular signals. In the following, all models based on BMs with classical coefficients will be denoted as “CBM” whereas all models based on BMs with revised coefficients will be referred to as “RBM” (compare Table 1). In short, the distinction between CBM and RBM rests on the substitution:

$$k_{1}=(1-V_{0})4.3\vartheta_{0}E_{0}TE\text{,}k_{2}=2E_{0}\text{,}k_{3}=1-\varepsilon \to k_{1}=4.3\vartheta_{0}E_{0}TEk_{2}=\varepsilon r_{0}E_{0}TEk_{3}=1-\varepsilon$$

#### Linear vs. non-linear BOLD equations

Over and above the non-linearities in the state equations for blood volume and deoxyhemoglobin content (Eq. (8)), the Buxton BM and our generalized BM have a non-linear form (Eqs. (2) and (12)) whereas the Obata BM is linear (Eq. (11)). The Obata BM can be regarded as a linearized version of the generalized BM which is due to a simplified approximation to the exact signal change equation (see Appendix B). Concerning the Buxton BM, the non-linearity is introduced by the intravascular term: *φ*(*q*,*ν*) = 1 − *q*/*ν*. In order to obtain a linearized version of it, it therefore suffices to complement the reduction of the coefficients described above with a linearization of this term around the resting state (*q* = *ν* = 1), using the first-order Taylor approximation; *φ*(*q*,*ν*) ≈ *ν* − *q*:

(13) $$\frac{\Delta S}{S_{0}}\approx V_{0}\left[k_{1}(1-q)+k_{2}\left(1-\frac{q}{v}\right)+k_{3}(1-v)\right]\to V_{0}[(k_{1}+k_{2})(1-q)+(k_{3}-k_{2})(1-v)]$$

Throughout this paper, linear and non-linear models are distinguished by the subscripts “L” and “N”, respectively. For example, the Obata model is referred to as “RBM_L_”, whereas the original Buxton model is referred to by “CBM_N_” (see Table 1).

#### Free vs. fixed parameterization of intra- and extravascular signals

A critical parameter in BM is *ε*, the ratio of intra- and extravascular signals. This quantity is not well characterized because it is difficult to disambiguate intra- and extravascular BOLD signal experimentally. Obata et al. (2004) estimated *ε* = 1.43 by assuming intra- and extravascular relaxation time constants of *T*_2I_* = 90 ms (corresponding to *R*_2I_* = 11.1 s^− 1 ^) and *T*_2E_* = 50 ms, respectively, and equal spin-densities in intra- and extravascular compartments. The exact basis of these assumptions is not quite clear. For the *T*_2I_* value, Obata et al. (2004) referred to the study by Li et al. (1998) who measured *T*_2_* values in large vessels of pigs. However, in their paper, Li et al. (1998) state: “... the *R*_2_* values of arterial (Y ≈ 93%) and venous (Y ≈ 75%) blood measured in our pig studies are approximately 4 and 6 s^− 1 ^, respectively, whereas those measured in human volunteers in our previous work are approximately 5 and 10 s^− 1 ^, respectively...” For their *T*_2E_* estimate, Obata et al. (2004) did not give a reference.

Overall, there is considerable uncertainty about the value of *ε* in the literature. In contrast to Obata et al. (2004), Buxton et al. (1998) assumed that *ε* = 0.4. Recent data from Lu and Van Zijl (2005), obtained with a “vascular space occupancy” technique that nulls intravascular contributions to the BOLD signal, suggest the contribution of extravascular signal to the total change in *R*_2_* during activation is 47% at 1.5 T. This implies that in their measurements *ε* ≈ 1. Furthermore, the results of Silvennoinen et al. (2006) imply that ε depends on complex interactions between TE and field strength.

**Fig. 2:**
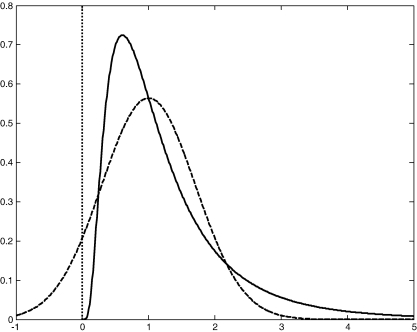
This figure shows a log-normal probability density function (solid line) with a mean of 1 and a variance of 0.5. For comparison, a Gaussian probability density function with identical mean and variance is shown (dashed line). Note that in contrast to the Gaussian, the support of the log-normal density is restricted to positive numbers, as indicated by the dotted vertical line.

This suggests that instead of fixing the value of *ε*, it may be more appropriate to acknowledge the uncertainty about it and make it a free parameter of the BM. Additionally, because different brain regions can differ in their vascularization and therefore in the intravascular contribution to the measured BOLD signal, different values of *ε* for each brain area in the DCM may be appropriate. The question remains however, how one should choose the prior density of *ε*. Since *ε* is the ratio of intra- and extravascular BOLD signal at rest, it is positive. This is assured with a log-normal prior density (see Fig. 2 ). Furthermore, given our uncertainty, this prior should be fairly flat. In our models, we used *ε* = *μ*_*ε*_ exp(*ϑ*_*ε*_), where the scale-parameter *ϑ*_*ε*_ had a prior mean of zero and a variance of 0.5. This allows for approximately an order of magnitude variation about *μ*_*ε*_; a range that is substantially larger than the estimates of ε described above. Furthermore, we chose *μ*_*ε*_ = 1 (i.e., equivalent intra- and extravascular signal contributions at rest). This is a value roughly in the middle of the *ε* estimates at 1.5 T reported so far (see above).

The distinction between treating *ε* as fixed or free parameter induces the final variant of the BMs we considered in this study. In this paper, a model in which *ε* is treated as a free parameter for each area is indicated by the suffix “(*ε*)”, whereas a model in which ε is a fixed parameter does not have a suffix (see Table 1).

## Methods

### Dynamic casual modeling (DCM)

DCM for fMRI uses a simple model of neural dynamics in a system of *n* interacting brain regions where neural population activity of each region is represented by a single state variable. It models the change of this neural state vector ***x*** in time as a bilinear differential equation:

(14) $$\frac{dx}{dt}=(A+\sum_{j=1}^{m}u_{j}B^{\left(j\right)})x+Cu$$

Here, the **A** matrix represents the fixed (context-independent or endogenous) strength of connections between the modeled regions, and the matrices **B** ^(*j*)^ represent the modulation of these connections (e.g., due to learning, attention, etc.) induced by the *j*th input *u*_*j*_ as an additive change. Finally, the **C** matrix represents the influence of direct (exogenous) inputs to the system (e.g., sensory stimuli). Note that all parameters are rate constants and are thus in units of s^−^^1^.

To explain regional BOLD responses, DCM for fMRI combines this model of neural dynamics with the hemodynamic model described above. Together, the neural and hemodynamic state equations yield a deterministic forward model with hidden states. For any given combination of parameters *θ* and inputs *u*, the measured BOLD response *y* is modeled as the predicted BOLD signal *h*(*u*,*θ*) plus a linear mixture of confounds *Xβ* (*e.g*., signal drift) and observation error *e*:

(15) y=h(u,θ)+Xβ+e

DCM uses a fully Bayesian approach to parameter estimation, with empirical priors for the hemodynamic parameters and conservative shrinkage priors for the coupling parameters (see Friston, 2002 and Friston et al. 2003 for details). Briefly, the posterior moments are updated iteratively using variational Bayes under a fixed-form Laplace (i.e., Gaussian) approximation, *q*(*θ*), to the conditional density *p*(*θ*|*y*). This includes a gradient ascent on a variational free-energy bound on the marginal likelihood to optimize the maximum a posteriori (MAP) estimate of the parameters in the E-step of an EM algorithm, whereas the M-step is concerned with optimizing hyperparameters *λ* that control the covariance components of observation error.

### Bayesian model selection (BMS)

Given some observed data, which of several alternative models is optimal? The decision cannot be made solely by comparing the relative fit of competing models. One also needs to account for differences in complexity; i.e., the number of free parameters and the functional form of the generative model (Pitt and Myung, 2002). This is important because as model complexity increases, fit increases monotonically, but at some point the model will start fitting noise that is specific to the particular data (*i.e.*, “over-fitting”) and thus becomes less generalizable across multiple realizations of the same underlying generative process. Therefore, the question “What is the optimal model?” can be reformulated as “What is the model that represents the best balance between fit and complexity?” This is the model that maximizes the model evidence:^4^

(16) p(y|m)=∫p(y|θ,m)p(θ|m)dθ

Here, the numbers of free parameters (as well as the functional form) are subsumed by the integration. Unfortunately, this integral cannot usually be solved analytically; therefore an approximation to the model evidence is used. This is usually the free-energy bound on the log-evidence (Friston et al., 2007). In DCM, the negative free energy *F* is the objective function for inversion:

(17) F=lnp(y|m)−KL[q(θ),p(θ|y,m)]

Here, KL denotes the Kullback–Leibler divergence between the approximating posterior density *q*(*θ*) and the true posterior *p*(*θ*|*y*,*m*) (Friston et al., 2007). After convergence of the estimation scheme, the KL term is minimized and *F* ≈ ln *p*(*y*|*m*). Rewriting Eq. (17) as

(18) $$F={\langle \log p(y|\theta \text{,}m)\rangle }_{q}-\mathrm{KL}(q(\theta )\text{,}p(\theta ))$$

adds a useful perspective, The two terms in Eq. (18) can be understood as encoding the two opposing requirements of a good model: that it explains the data and is as simple as possible (i.e., uses minimal number of parameters that deviate minimally from their priors). Eqs. (17) and (18) are derived in Appendix A.

To quantify the relative goodness of two models *m*_*i*_ and *m*_*j*_, the differences in their log-evidences can be transformed into a Bayes factor (BF):

(19) $${\mathrm{BF}}_{ij}=\frac{p(y|m_{i})}{p(y|m_{j})}\approx \exp(F_{i}-F_{j})$$

The group Bayes factor (GBF) for any given model *m*_*i*_, relative to *m*_*j*_, is

(20) $${\mathrm{GBF}}_{ij}=\exp\left(\sum_{k}F_{i}-F_{j}\right)=\prod_{k}{\mathrm{BF}}_{ij}^{k}$$

where *k* indexes subjects. As detailed in Stephan and Penny (2007), the GBF is equivalent to the product of the subject-specific Bayes factors for a given model comparison. It rests on the assumption that model evidences are independent across subjects; this is tenable if the subjects are statistically independent samples from the population. Furthermore, given a flat prior on the model, the product of model evidences is equivalent to multiplying the posterior probabilities of all models.

Just as conventions have developed for using *p*-values in frequentist statistics, there are conventions for Bayes factors. For example, Raftery (1995) suggests an interpretation of Bayes factors as providing weak (BF < 3), positive (3 ≤ BF < 20), strong (20 ≤ BF < 150), or very strong (BF ≥ 150) evidence of one model over another. A complementary index to the GBF is the *positive evidence ratio* (PER; Stephan and Penny, 2007), i.e., the number of subjects where there is positive (or stronger) evidence for model *m*_*i*_ divided by the number of subjects with positive (or stronger) evidence for model *m*_*j*_

(21) $${\mathrm{PER}}_{ij}=\frac{\left|k:BF_{ij}^{k}>3\right|}{\left|k:BF_{ji}^{k}>3\right|}$$

where *k* = 1,…,*N* and ∣·∣ denotes set size. The GBF is sensitive to outliers, whereas the PER is not. In contrast, the PER is insensitive to the magnitude of the differences across subjects while the GBF is not. Therefore, the GBF and the PER can be used as complementary measures: the GBF gives a quantitative account of the difference, pooling over all data (i.e., subjects) but ignoring inter-subject variability, whereas the PER describes the qualitative reproducibility of model comparison over subjects.

### Using DCM and BMS to evaluate different hemodynamic models

**Fig. 3:**
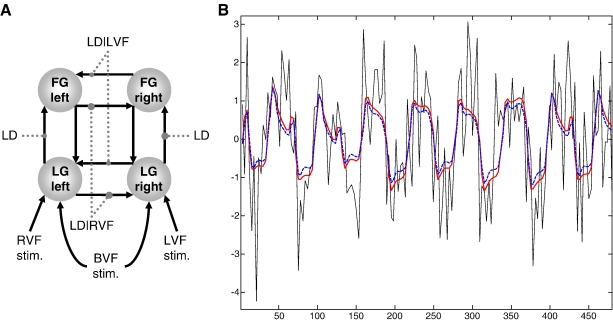
(A) Summary of the DCM used in this evaluation study (see Stephan et al., 2007a for details). This is a four-area model, comprising reciprocally connected lingual gyrus (LG) and fusiform gyrus (FG) in both hemispheres. Non-foveal visual stimuli (words) were presented in either the right (RVF) or left (LVF) visual field with a randomized stimulus onset asynchrony between 1.5 and 2.5 s during 24 s blocks; these were modeled as individual events driving contralateral LG activity. During the instruction periods, bilateral visual field (BVF) input was provided for 6 s; this was modeled as a box-car input to LG, in both hemispheres. Connections were modulated by task and stimulus properties (grey dotted lines). Intra-hemispheric LG→FG connections were allowed to vary during a letter decision (LD) task, regardless of visual field. In contrast, inter-hemispheric connections were modulated by task conditional on the visual field (LD|LVF and LD|RVF). (B) This figure provides an anecdotal example of how two different models (red solid line: RBM_N_(*ε*); blue dashed line: RBM_L_) fit measured BOLD data (black solid line). For this example, we chose the first 160 scans from the left fusiform gyrus in a single subject from this study. The *x*-axis denotes seconds, *y*-axis denotes percent signal change.

We compared the variants of hemodynamic models described above by placing them in a DCM and comparing the resulting models using BMS. We used a well-characterized DCM of interacting visual areas as a vehicle for this comparison. This DCM is the four-area ventral stream model described by Stephan et al. (2007a), which had emerged as the optimal model from a systematic comparison of sixteen alternatives. Fig. 3A shows the structure of this model. The fMRI data are from the study by Stephan et al. (2003) and were obtained at 1.5 T and a TE of 66 ms. The advantage of using a full DCM (instead of modeling a single area as in Friston, 2002) is twofold: first, the resulting inference does not depend on the choice of a particular region, and second, the neural state equation in DCM can model a wide range of neuronal transients, sustained responses, and adaptation effects and thus affords much higher realism than other hemodynamic models (see Discussion). Furthermore, our comparisons were performed separately for each of 12 subjects, to avoid conclusions that were specific to a particular individual.

**Fig. 4:**
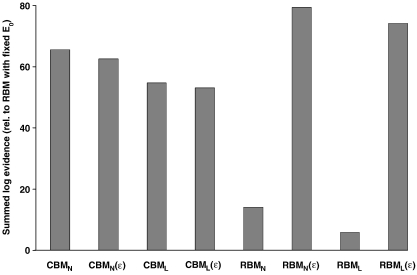
Summary of the model comparison results. This graph compares the eight DCMs resulting from the factorial structure of our comparison (classical vs. revised coefficients, linear vs. non-linear BOLD equations, fixed *ε* vs. free *ε*) against a reference model (the original revised model by Obata et al., 2004, with fixed values for *E*_0_ and *ε*.). The figure shows the log of the group Bayes factor (i.e., the log model evidence summed across the twelve subjects, minus the summed log-evidence for the reference model). Except for the reference model, all models treated *E*_0_ as a free parameter. See Table 1 for interpretation of the model names.

Our model comparison adopted a factorial approach: we compared (i) classical vs. revised forms for the coefficients, (ii) linear vs. non-linear BMs, and (iii) fixed vs. free *ε*. All combinations were tested so that we could establish the relative importance of the three model attributes. This resulted in eight DCMs per subject. As an additional reference model for displaying the log-evidences, we chose the model of Obata et al. (2004) in its original implementation, where both ε and resting oxygen extraction fraction, *E*_0_ have a fixed value. In our experience, treating *E*_0_ as a free parameter typically improves the evidence of hemodynamic models. Therefore, all of our eight models treated *E*_0_ as a free parameter. Indeed, model comparison showed that all eight models were superior to the reference model (see Fig. 4 ).

## Results

The same DCM, equipped with different BOLD output equations, was fitted to the empirical fMRI data from each subject. Fig. 4 summarizes the results of all model comparisons. For each model, it shows the log of the GBF; i.e., the sum of its log-evidences across the group minus the summed log-evidence for the reference model (cf. Eq. (20)). It can be seen that the best of all models is the non-linear model with revised coefficients and free ε, i.e., RBM_N_(*ε*). In the following, we describe the individual comparisons in more detail. We first report the direct comparison between the classical Buxton model (CBM_N_) and the Obata model (RBM_L_). These two models differ in terms of both reduction (of the coefficients) and linearization; therefore we then investigate whether any differences depend on treating *ε* as a free or a fixed parameter. Finally, we examine the role of non-linear vs. linear BOLD models.

### Comparing the Buxton model (CBM) vs. the Obata model (linear RBM)

First, we investigated the effect of freeing E_0_ in the Obata model. Our implementation, which treated *E*_0_ as a free parameter (i.e., RBM_L_), was slightly superior to the original formulation by Obata et al. (2004) in which *E*_0_ had a fixed value (GBF = 3.49 × 10^2^; PER = 1:0; second column of Table 2 ). Note that the latter is used as a reference model for plotting the results in Fig. 4. In a next step, we compared the linear RBM with the classical Buxton model (CBM_N_). This comparison indicated that CBM_N_ was superior in each and every subject (PER = 12:0), giving a GBF of 8.41 × 10^25^ (Table 2, third column). We next investigated the two possible reasons why RBM_L_ performed worse than CBM_N_: (i) a suboptimal value of ε, and/or (ii) the failure to model non-linearities in the BOLD equation.

### Fixed vs. free *ε*

For the linear RBM_L_, freeing ε dramatically improved the model evidence: RBM_L_ was clearly inferior to RBM_L_(ε) (GBF = 2.12 × 10^− 30 ^; PER = 0:12; Table 2, fourth column). RBM_L_(*ε*) also performed better, albeit only slightly, than CBM_N_ (GBF = 5.60 × 10^3^, PER = 2:1).

In contrast, for CBM_N_, freeing ε did not improve it and actually slightly decreased its log-evidence: CBM_N_(*ε*) performed worse than the original CBM_N_ with fixed *ε* (GBF = 5.24 × 10^− 2 ^, PER = 1:3). These comparisons illustrate that å plays a different role within the linear RBM_L_(*ε*) and the non-linear CBM_N_(*ε*), respectively: in the latter, including *ε* as an additional free parameter per region led to an increase in model complexity (by increasing the overall number of model parameters and changing how other parameters had to deviate from their priors) that outweighed the improvement in data fit, whereas in the former it did not. Would the same results also hold for the linear CBM_L_(*ε*) and the non-linear RBM_N_(*ε*)?

### The influence of non-linearities in the BOLD equation

Fitting the linear CBM_L_ to the data and evaluating its log-evidence showed that it performed worse than the other CBM variants described above (see Fig. 4). With fixed ε, although it was still clearly superior to the linear RBM_L_ (GBF = 1.57 × 10^21^, PER = 12:0), it was not as good as the original CBM_N_ (GBF = 1.87 × 10^−^^5^, PER = 0:3), and the linear RBM_L_(*ε*) (GBF = 3.34 × 10^− 9 ^, PER = 0:3). Freeing *ε* further decreased its log-evidence: the linear CBM_L_(*ε*) turned out to be the worst of all CBM variants tested here (see Fig. 4).

When the non-linear RBM_N_ was evaluated using BMS, using a fixed *ε*, it was superior to the linear RBM_L_ with fixed *ε* (GBF = 3.63 × 10^3^; PER = 3:0), but inferior to the CBM_N_ (GBF = 4.32 × 10^−23^; PER = 0:11) (see Table 3 ). However, when *ε* was treated as a free parameter in RBM_N_, it became superior to all other models (see Fig. 4). It even performed slightly better than the previously best model, the linear RBM_L_(*ε*) (GBF = 1.92 × 10^2^). However, examination of the subject-specific BFs in Table 3 (rightmost column) shows that this improvement is fairly subtle: the individual BFs are only in the range 0.97 to 2.32. This indicates that the differences are very small at the level of individual subjects. However, given the consistent direction of the individual BFs over the group, the GBF reflects clear evidence in favor of the non-linear RBM_N_(*ε*). In summary, freeing *ε* decreased the goodness of both linear and non-linear CBMs, but improved both linear and non-linear RBMs, with the non-linear RBM_N_(ε) emerging as the best model from our comparisons.

### Analysis of posterior covariances and robustness of connectivity estimates

An interesting issue is whether estimates of the new parameter *ε* allow for straightforward interpretation, or whether this parameter exhibits conditional dependencies with other parameters. The region-specific parameter estimates for *ε*, averaged across subjects, were 0.63 ± 0.43 (left lingual gyrus, LG), 0.58 ± 0.36 (right LG), 1.98 ± 0.45 (left fusiform gyrus, FG), and 1.38 ± 0.46 (right FG). It is striking that for the regions receiving direct inputs (left and right LG; compare Fig. 3A) the estimates are lower than the prior mean of *ε*. In contrast, for the regions not receiving direct inputs (left and right FG), they are higher.

**Fig. 5:**
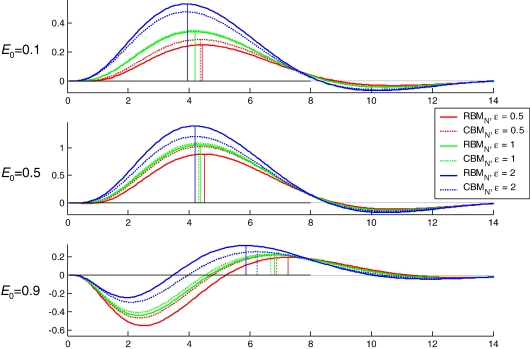
This figure demonstrates the effect that changing the resting oxygen extraction fraction, *E*_0_, and the ratio of intra- to extravascular BOLD signal at rest, *ε*, has on the shape and amplitude of the hemodynamic impulse response as generated by the RBM_N_ (solid lines) and CBM_N_ (dotted lines) models. Colors indicate different values of *ε* (see legend). Vertical lines indicate the position of the response maximum.

**Fig. 6:**
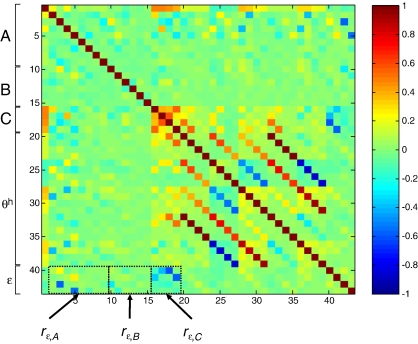
Posterior correlation matrix for the RBM_N_(*ε*), averaged across all twelve subjects. Three correlations are of particular interest (see black rectangles). *ε* shows negative correlations (up to − 0.61; *r*_*ε,*C _) with the input parameters, C. A small subset of the A parameters (fixed connection strengths) also correlate negatively with *ε* (up to − 0.53; *r*_*ε,*A _). Importantly, *ε* is not strongly correlated with the parameters of main interest within DCM, i.e., with the parameters of the context-dependent modulation of connections, B. These correlations were mostly close to zero and maximally − 0.29 (*r*_*ε,*B _). *θ*^h^ = hemodynamic parameters (except *ε*).

As demonstrated in Fig. 5 , increasing ε increases the amplitude of the evoked BOLD response (although note that the effect of changing å depends on the value of *E*_0_). This effect is similar to that achieved by increasing the driving inputs (**C** in Eq. (14)). In order to fit a given BOLD response, any change in *ε* or **C**, respectively, would therefore have to be compensated, to some degree, by changing the other parameter in the opposite direction. This means that the two parameters are likely to show inverse conditional correlations. This was confirmed by an analysis of the estimated posterior covariances of the parameters in the RBM_N_(*ε*) model. Since covariance matrices are difficult to interpret visually, we normalized the posterior covariance matrix from each subject to provide a posterior correlation matrix. Fig. 6 shows the average posterior correlation matrix over subjects. The rectangles in this figure highlight three results: first, *ε* shows a fairly strong negative correlation with the input parameters to the system, **C** (see *r*_*ε,***C**_ in Fig. 6). As expected, this posterior correlation was particularly pronounced (up to − 0.61) for the *ε*’s in the regions that received driving inputs (i.e., left and right lingual gyrus, LG). Second, *ε* also shows a negative correlation (up to − 0.53) with the fixed strengths (**A** in Eq. (14)) of those connections that convey activity elicited in input areas (left and right LG) to those areas not receiving direct inputs (left and right FG). These connections play a similar role in determining the amplitude of the BOLD response in left and right FG as do the driving inputs for left and right LG. Most importantly, however, *ε* is *not* strongly correlated with the main parameters of interest within DCM, that is, with the parameters encoding the context-dependent modulation of connection strengths (**B** in Eq. (14)). These correlations did not go beyond − 0.29 and were mostly close to zero (see *r*_*ε,***B**_ in Fig. 6).

Tables 4 and 5 show how a change in the hemodynamic model affects the average neuronal parameter estimates and their standard error across subjects. It can be seen that in models with free ε the conditional dependencies between *ε* and the neuronal parameters described above have only modest effects on the parameter estimates. As expected, the changes are most profound for the **C** parameters (Table 5), which are usually not of interest. Concerning the **A**/**B** parameters (Table 4), in no case were differences induced by freeing ε large enough to affect second-level statistical inference about the parameter estimates^5^ . Finally, as can be seen in the tables, other changes in the hemodynamic model affect the parameter estimates even less than freeing *ε*.

## Discussion

In this study, we implemented a variety of different BOLD signal models within the hemodynamic forward model of DCM. We chose an established four-area DCM of the visual cortex (Stephan et al. 2007a) which was fitted to empirical fMRI data from 12 subjects. The relative goodness of the different BOLD signal models was assessed by means of BMS. This comparison gave three main results. First, we showed that, in its original formulation, the linear RBM_L_ (which was recently introduced by Obata et al., 2004) is not superior to the CBM_N_ (the classical BM by Buxton et al., 1998). Second, we have demonstrated that RBM_L_ can be made to outperform the CBM_N_ if ε, the ratio between intra- and extravascular signal components, is allowed to vary as a free parameter. Third, we have derived a generalized non-linear BM with revised coefficients that outperforms any other model tested, when *ε* is treated as a free parameter (the RBM_N_(*ε*) variant in this study). This model was founded on the previous observation (Friston et al., 2000) that a non-linear output equation improves modeling of the BOLD signal. Because of its superior performance in this study, this hemodynamic model will replace the CBM in the next update of the DCM routines in the open source software package SPM5 (http://www.fil.ion.ucl.ac.uk/spm). The other hemodynamic models tested here will be included as options, allowing users to compare alternatives and find the most appropriate hemodynamic model for their particular experimental set-up.

The posterior covariances among parameters suggest that most parameters of interest in a DCM, i.e., the connectivity among regions and particularly its context-dependent modulation (*A* and *B* matrices in Eq. (14)), are not strongly correlated with the hemodynamic parameters, including the new parameter ε (Fig. 6). This relative independence of the neuronal and hemodynamic parameters is an important feature of DCM, which largely results from normalizing the coupling parameters with regard to the system’s dampening rate-constant (i.e., the diagonal of the **A** matrix; see Friston et al., 2003 for details). This renders them less dependent on the amplitude of the hemodynamic response and the neuronal input parameters (**C** in Eq. (14)). If the two sets of parameters were strongly coupled, it could be difficult to disambiguate the influence of neuronal connectivity from neurovascular coupling mechanisms on the resulting BOLD response. Tables 4 and 5 lists all mean parameter estimates in our model and their standard error across subjects for each of the hemodynamic models tested in this study. It can be seen that in no case changes in coupling parameters were large enough that the nature of statistical inference (as obtained by a second-level *t*-test) would have changed.

From a broader perspective, these results suggest that the particular form of the hemodynamic model is not terribly important for inference about the underlying neuronal dynamics and how they were caused. Although one might have intuited this from the rather simple form of empirically derived hemodynamic impulse response functions, it is reassuring to see that the specific parameterizations employed by the biophysical models in DCM provide inference on neuronal parameters that are robust to changes or misspecification of the hemodynamic model. Although the nature of the hemodynamic model may not affect inferences at the neuronal level, it is clearly important for inference on the hemodynamic parameters that may be of interest when studying regional variations in physiology or pathophysiology *per se*.

Our characterization of conditional dependencies among the parameters is related to measures of system identifiability. Identifiability can be addressed from various angles. For example, sensitivity analyses investigate the partial derivatives of model output with regard to the parameters. A system is not identifiable if, for any change of a given parameter, an identical effect on model output can be achieved by changing one or several other parameters. In Bayesian approaches to system identification, conventional sensitivity analyses and analyses of posterior covariances have a close relationship. An example of this was given by Deneux and Faugeras (2006) who performed a sensitivity analysis for the hemodynamic model of Buxton et al. (1998), deriving a sensitivity factor that was proportional to the inverse of the posterior covariance of the parameters (under the Laplace approximation this inverse is also known as Fishers Information matrix). We used the conditional covariances directly (after normalizing them to form conditional correlation matrices). These matrices encode the degree of interdependence between the parameters (see Fig. 6).

Several considerations should be kept in mind when interpreting the results of this study. First, the results were obtained using a particular data set and BOLD time-series from visual cortex. Even though we used four different visual areas and replicated the results in twelve subjects, we cannot ensure that the optimal model is invariant across brain regions and data sets.

Second, the various hemodynamic models in this study were investigated as an integral part of DCM, a causal model that links experimentally designed manipulations (e.g., presentation of sensory stimuli) via predicted neural and vascular dynamics to observed BOLD responses of discrete brain areas. Although this model is fairly abstract at the neural level, representing neuronal population activity by a single state variable for each region, it allows for a much more flexible representation of neural responses to external perturbations than other hemodynamic models available. For example, it is not constrained to adaptation effects of a particular form as in Buxton et al. (2004), but can represent any kind of neural dynamics that can be modeled with bilinear differential equations. This comprises a wide range of transients, sustained responses and adaptation effects (see Fig. 1 in Penny et al., 2004b and Fig. 5 in Stephan et al., 2007b for examples).

Third, the mathematical form of the dependence of the BOLD signal on deoxyhemoglobin content *q* and blood volume *v* remains an interesting research question. Several previous studies suggested that the CBF–BOLD relation has significant non-linear components (Birn et al., 2001; Deneux and Faugeras, 2006; Friston et al., 2000; Miller et al., 2001; Vazquez and Noll, 1998; Wager et al., 2005), and the work presented here supports this view. These non-linearities could enter the system at various levels of the biophysical process. In fact, the state equations for both *q* and *ν* are non-linear (although, given the typical range of values for *q* and *ν*, these non-linearities are rather weak). Thus, even a model with a linear BOLD equation as, for example, the model by Obata et al. (2004) can show a non-linear CBF–BOLD relationship. However, in agreement with Friston et al. (2000), the present results suggest that the output non-linearity of the Balloon model is important, over and above the non-linearity of the state equations for *q* and *ν*. The reason for this is that the estimation scheme in DCM uses a bilinear approximation to the states while retaining the output non-linearity (cf. Friston et al. 2003).

Fourth, the hemodynamic models in the present study assumed a field strength of 1.5 T. The finding that the neuronal connectivity parameters are fairly robust to changes in the hemodynamic output equation (Table 4), suggests that the current implementation of DCM can also be used in situations for which the coefficients in the hemodynamic model are not optimized (e.g., field strengths higher than 1.5 T). It should also be possible in the future, however, to adapt the model to higher field strengths since most constants in Eq. (12) are known or can be computed for higher field strengths. The main problem is *ε* for which, to our knowledge, there is a lack of reliable empirical estimates, particularly at high field strengths. However, the present study demonstrated that one can treat *ε* as a free parameter, and using a flat prior for this parameter properly accommodates the uncertainty about its value.

Finally, the importance of non-linearity in the BOLD output equation depends on the exact stimulation conditions; in particular it is likely to increase with shorter stimulus-onset asynchronies (SOA) than the ones used in this study (1.5–2.5 s) and to decrease with longer SOAs (Friston et al., 1998). In any case, the procedure we introduced here (and the code that will be made available in SPM5) enables the user to examine this question for her or his specific data, by comparing different hemodynamic models.

## Figures and Tables

**Table 1:** This table summarizes the nomenclature used to describe the eight different models compared in this study

Model name	Coefficients	BOLD equation	*ε*
CBM_N_	Classical (Buxton et al., 1998)	Non-linear	Fixed
CBM_N_(ε)	Classical (Buxton et al., 1998)	Non-linear	Free
CBM_L_	Classical (Buxton et al., 1998)	Linear	Fixed
CBM_L_(ε)	Classical (Buxton et al., 1998)	Linear	Free
RBM_N_	Revised (Obata et al., 2004)	Non-linear	Fixed
RBM_N_(ε)	Revised (Obata et al., 2004)	Non-linear	Free
RBM_L_	Revised (Obata et al., 2004)	Linear	Fixed
RBM_L_(ε)	Revised (Obata et al., 2004)	Linear	Free

**Table 2:** This table shows the individual Bayes factors, for each of the twelve subjects in the group studied, for selected comparisons of the RBM_L_ model against other model variants

Subjects	RBM_L_ vs. reference	RBM_L_ vs. CBM_N_	RBM_L_ vs. RBM_L_(*ε*)
1	3.78E+ 00	8.66E− 02	4.34E− 02
2	1.35E+ 00	7.63E− 04	3.25E− 03
3	1.33E+ 00	2.17E− 03	3.62E− 03
4	1.65E+ 00	1.25E− 04	1.04E− 08
5	1.14E+ 00	1.51E− 03	1.48E− 03
6	1.05E+ 00	1.52E− 04	1.52E− 04
7	1.62E+ 00	5.28E− 02	8.77E− 02
8	1.43E+ 00	1.70E− 03	3.39E− 04
9	2.38E+ 00	1.16E− 01	1.27E− 01
10	1.79E+ 00	2.46E− 01	2.49E− 01
11	1.47E+ 00	2.26E− 02	1.59E− 02
12	1.80E+ 00	5.01E− 02	1.19E− 01
Group BF	3.49E+ 02	1.19E− 26	2.12E− 30
PER	1:0	0:12	0:12

BF = Bayes factor, PER = positive evidence ratio.

**Table 3:** This table shows the individual Bayes factors, for each of the twelve subjects in the group studied, for selected comparisons of the non-linear RBM_N_ model

Subjects	RBM_N_ vs. RBM_L_	RBM_N_ vs. CBM_N_	RBM_N_(*ε*) vs. RBM_L_(*ε*)
1	8.34E− 01	7.23E− 02	9.76E− 01
2	3.19E+ 00	2.44E− 03	1.96E+ 00
3	2.08E+ 00	4.50E− 03	1.59E+ 00
4	4.02E+ 00	5.01E− 04	2.18E+ 00
5	1.73E+ 00	2.61E− 03	1.42E+ 00
6	3.82E+ 00	5.82E− 04	2.32E+ 00
7	2.04E+ 00	1.08E− 01	1.67E+ 00
8	1.36E+ 00	2.31E− 03	1.17E+ 00
9	1.69E+ 00	1.97E− 01	1.48E+ 00
10	1.38E+ 00	3.38E− 01	1.21E+ 00
11	1.28E+ 00	2.89E− 02	1.19E+ 00
12	2.98E+ 00	1.49E− 01	2.12E+ 00
Group BF	3.63E+ 03	4.32E− 23	1.92E+ 02
PER	3:0	0:11	0:0

BF = Bayes factor, PER = positive evidence ratio.

**Table 4:** This table shows the mean parameter estimates across subjects (± standard error) for the fixed connection strengths (A) and their context-dependent modulation (B), from all eight hemodynamic models

	LGl→LGr	LGl→FGl	LGr→LGl	LGr→FGr	FGl→LGl	FGl→FGr	FGr→LGr	FGr→FGl
*Fixed connection strengths (A)*								
CBM_N_	0.25 ± 0.05	0.31 ± 0.05	0.18 ± 0.07	0.25 ± 0.03	0.24 ± 0.06	0.10 ± 0.04	0.12 ± 0.04	0.07 ± 0.07
CBM_N_(*ε*)	0.25 ± 0.05	0.32 ± 0.04	0.19 ± 0.07	0.24 ± 0.03	0.25 ± 0.07	0.08 ± 0.04	0.10 ± 0.04	0.07 ± 0.07
CBM_L_	0.25 ± 0.05	0.32 ± 0.05	0.18 ± 0.07	0.25 ± 0.03	0.24 ± 0.06	0.10 ± 0.03	0.12 ± 0.04	0.07 ± 0.07
CBM_L_(ε)	0.25 ± 0.05	0.30 ± 0.04	0.18 ± 0.07	0.24 ± 0.03	0.25 ± 0.07	0.08 ± 0.04	0.11 ± 0.04	0.07 ± 0.07
RBM_N_	0.27 ± 0.05	0.33 ± 0.05	0.21 ± 0.07	0.26 ± 0.03	0.24 ± 0.06	0.09 ± 0.04	0.11 ± 0.04	0.07 ± 0.07
RBM_N_(*ε*)	0.23 ± 0.06	0.24 ± 0.03	0.19 ± 0.06	0.21 ± 0.03	0.23 ± 0.07	0.06 ± 0.04	0.09 ± 0.04	0.05 ± 0.06
RBM_L_	0.27 ± 0.05	0.33 ± 0.05	0.21 ± 0.07	0.26 ± 0.03	0.24 ± 0.06	0.10 ± 0.04	0.12 ± 0.04	0.07 ± 0.07
RBM_L_(*ε*)	0.23 ± 0.06	0.25 ± 0.04	0.19 ± 0.06	0.21 ± 0.03	0.23 ± 0.07	0.06 ± 0.04	0.09 ± 0.04	0.05 ± 0.06

*Context-dependent modulations (B)*								
CBM_N_	0.03 ± 0.02	0.35 ± 0.06	0.25 ± 0.04	0.17 ± 0.05	NA	0.02 ± 0.02	NA	0.12 ± 0.03
CBM_N_(*ε*)	0.04 ± 0.03	0.33 ± 0.05	0.25 ± 0.04	0.16 ± 0.05	NA	0.02 ± 0.02	NA	0.10 ± 0.03
CBM_L_	0.03 ± 0.02	0.35 ± 0.06	0.25 ± 0.04	0.17 ± 0.05	NA	0.02 ± 0.02	NA	0.13 ± 0.03
CBM_L_(*ε*)	0.03 ± 0.03	0.33 ± 0.06	0.25 ± 0.04	0.17 ± 0.05	NA	0.02 ± 0.02	NA	0.11 ± 0.03
RBM_N_	0.03 ± 0.02	0.35 ± 0.06	0.24 ± 0.04	0.17 ± 0.05	NA	0.02 ± 0.02	NA	0.12 ± 0.03
RBM_N_(*ε*)	0.05 ± 0.03	0.32 ± 0.04	0.26 ± 0.04	0.15 ± 0.05	NA	0.02 ± 0.02	NA	0.09 ± 0.03
RBM_L_	0.03 ± 0.03	0.35 ± 0.06	0.24 ± 0.04	0.17 ± 0.05	NA	0.02 ± 0.02	NA	0.12 ± 0.03
RBM_L_(*ε*)	0.05 ± 0.03	0.32 ± 0.04	0.26 ± 0.04	0.15 ± 0.05	NA	0.02 ± 0.02	NA	0.09 ± 0.03

It can be seen that the parameter estimates are fairly stable across models. Abbreviations of areas: LGl = left lingual gyrus; LGr = right lingual gyrus; FGl = left fusiform gyrus; FGr = right fusiform gyrus. NA = non-applicable. See Fig. 3A for the structure of the DCM.

**Table 5:** This table shows the mean estimates across subjects (± standard error) for the input parameters (C), from all eight hemodynamic models

	LVF→LGr	RVF→LGl	BVF→LGl	BVF→LGr
CBM_N_	0.15 ± 0.03	0.15 ± 0.04	0.19 ± 0.03	0.17 ± 0.02
CBM_N_(*ε*)	0.13 ± 0.03	0.14 ± 0.04	0.17 ± 0.02	0.16 ± 0.02
CBM_L_	0.14 ± 0.02	0.14 ± 0.04	0.18 ± 0.02	0.16 ± 0.02
CBM_L_(*ε*)	0.13 ± 0.02	0.13 ± 0.04	0.16 ± 0.02	0.15 ± 0.02
RBM_N_	0.10 ± 0.02	0.10 ± 0.03	0.13 ± 0.02	0.11 ± 0.01
RBM_N_(*ε*)	0.15 ± 0.03	0.15 ± 0.04	0.18 ± 0.02	0.18 ± 0.02
RBM_L_	0.10 ± 0.02	0.10 ± 0.03	0.12 ± 0.01	0.10 ± 0.01
RBM_L_(*ε*)	0.15 ± 0.03	0.14 ± 0.04	0.18 ± 0.02	0.18 ± 0.02

Proportionately, these estimates vary more strongly than those of the A/B parameters in Table 4. Abbreviations of inputs: LVF = left visual field input; RVF = right visual field input; BVF = bilateral visual field input. Abbreviations of areas: LGl = left lingual gyrus; LGr = right lingual gyrus; FGl = left fusiform gyrus; FGr = right fusiform gyrus. See Fig. 3A for the structure of the DCM.

## Appendix A Negative free energy as a lower bound on the log model evidence

The relation of the negative free energy *F* to the log model evidence, log *p*(*y*|*m*), can be derived by using an (arbitrary) approximating posterior density *q*(*θ*) to decompose *p*(*y*|*m*) into two components, i.e., *F* and the Kullback–Leibler divergence (KL) between the true posterior *p*(*y*|*m*) and the approximating posterior *q*(θ):

(A1) logp(y|m)=∫q(θ)logp(y|m)dθ

$$=\int q(\theta )\log\frac{p(y\text{,}\theta |m)q(\theta )}{p(\theta |y\text{,}m)q(\theta )}d\theta$$

$$=\int q(\theta )\log\frac{p(y\text{,}\theta |m)}{q(\theta )}d\theta +\int q(\theta )\log\frac{q(\theta )}{p(\theta |y\text{,}m)}d\theta$$

=F+KL[q(θ),p(θ|y,m)]

The KL divergence is an asymmetric measure of the differences between two probability densities (Kullback and Leibler, 1951). If the approximating posterior matches the true posterior density precisely, then KL[*q*(*θ*),*p*(*θ*|*y*,*m*)] = 0. This demonstrates that the negative free energy *F* is a lower bound on the log-evidence and can therefore be used as a criterion for model comparison. This makes the assumption that the KL divergence term is not drastically different across models (i.e., the tightness of the bound is similar under different models). For models like ours, with informed priors that lead to well-behaved posterior densities, this assumption is unlikely to be a strong one.

*F* itself can be decomposed into two components:

(A2) $$F=\int q(\theta )\log\frac{p(y\text{,}\theta |m)}{q(\theta )}d\theta$$

$$=\int q(\theta )\log\frac{p(y|\theta \text{,}m)p(\theta |m)}{q(\theta )}d\theta$$

$$=\int q(\theta )\log p(y|\theta \text{,}m)d\theta +\int q(\theta )\frac{p(\theta |m)}{q(\theta )}d\theta$$

$$={\langle \log p(y|\theta \text{,}m)\rangle }_{q}-\mathrm{KL}[q(\theta )\text{,}p(\theta )]$$

Here, the first term denotes the expected log likelihood (with regard to *q*) and thus describes the accuracy of the model in fitting the data (*i.e*., the goodness of fit). The second term describes how much the approximating posterior diverges from the prior density. This term is sensitive to the number of parameters and the form of the densities and can be regarded as a measure of model complexity. Together, this illustrates that *F* has properties that are required for model selection (see Pitt and Myung, 2002).

## Appendix B Derivation of a non-linear BOLD equation with revised coefficients

The revision of the Balloon model as suggested by Obata et al. (2004) led to a linear BOLD equation. This linear form results from several approximations during its derivation (see the appendix in Obata et al., 2004). Here, we start from the same point as in Obata et al. (2004) but use a first-order approximation to the exact BOLD signal equation without prior simplifications. This consistent approach to linearizing the model eventually preserves the non-linearity of the intravascular term.

Following Obata et al. (2004), we start by modeling the BOLD signal at rest, *S*_0_, as a volume-weighted sum of extra- and intravascular BOLD signals, *S*_E_ and *S*_I_ (*V*_0_ is the resting venous blood volume fraction):

(B1) $$S_{0}=(1-V_{0})S_{\text{E}}+V_{0}S_{\text{I}}$$

Given effective spin densities *S*_I0_ and *S*_E0_ and resting transverse relaxation rate constants *R*_2I_* and *R*_2E_* for the intra- and extravascular spaces, the two signal components can be modeled as:

(B2) $$S_{\text{E}}=S_{\text{E}0}\exp(-R_{2\text{E}}^{*}TE)$$

$$S_{\text{I}}=S_{\text{I}0}\exp(-R_{2\text{I}}^{*}TE)$$

With activation, the rate constants are changed by additive amounts Δ*R*_2I_* and Δ*R*_2E_*, resulting in a BOLD signal *S*:

(B3) $$S=(1-V)S_{\text{E}0}\exp[-(R_{2\text{E}}^{*}+\Delta R_{2\text{E}}^{*})TE]+VS_{\text{I}0}\exp[-(R_{2\text{I}}^{*}+\Delta R_{2\text{I}}^{*})TE]$$

$$=(1-V)S_{\text{E}}\exp(-\Delta R_{2\text{E}}^{*}TE)+VS_{\text{I}}\exp(-\Delta R_{2\text{I}}^{*}TE)$$

Using the relation *V* = *vV*_0_, where v is the venous blood volume fraction normalized with regard to rest, we can rewrite Eq. (B3) as:

(B4) $$S=(1-vV_{0})S_{\text{E}}\exp(-\Delta R_{2\text{E}}^{*}TE)+vV_{0}S_{\text{I}}\exp(-\Delta R_{2\text{I}}^{*}TE)$$

This is an exact expression for the BOLD signal *S* at activation. One can see that for a given *TE* and *V*_0_, it is a function of three quantities: Δ*R*_2E_*, Δ*R*_2I_*,*ν*. We now approximate the change in BOLD signal from rest to activation, Δ*S*, by a first-order Taylor expansion around the resting state (i.e., Δ*R*_2E_* = 0,Δ*R*_2I_* = 0,*ν* = 1; note that S_0_ is a constant and thus disappears during differentiation):

(B5) $$\Delta S=S-S_{0}$$

$$\Delta S\approx (v-1)\frac{\partial S}{\partial v}+\Delta R_{2\text{E}}^{*}\frac{\partial S}{\partial \Delta R_{2\text{E}}^{*}}+\Delta R_{2\text{I}}^{*}\frac{\partial S}{\partial \Delta R_{2\text{I}}^{*}}$$

$$=(v-1)(-S_{\text{E}}V_{0}+V_{0}S_{\text{I}})-\Delta R_{2\text{E}}^{*}(1-V_{0})S_{\text{E}}TE-\Delta R_{2\text{I}}^{*}V_{0}S_{\text{I}}TE$$

This is a consistent first-order approximation to the exact equation of BOLD signal change.

Noting that *ε* = *S*_I_/*S*_E_ and $\frac{1-V_{0}}{V_{0}}\approx \frac{1}{V_{0}}$ for a small venous blood volume fraction *V*_0_, one obtains:

(B6) $$\Delta S\approx (v-1)[V_{0}(S_{\text{I}}-S_{\text{E}})]-\Delta R_{2\text{E}}^{*}(1-V_{0})S_{\text{E}}TE-\Delta R_{2\text{I}}^{*}V_{0}S_{\text{I}}TE\text{,}$$

$$\approx V_{0}S_{\text{E}}\left[(v-1)(\varepsilon -1)-\frac{\Delta R_{2\text{E}}^{*}TE}{V_{0}}-\varepsilon \Delta R_{2\text{I}}^{*}TE\right]\text{,}$$

$$=S_{\text{E}}[(V-V_{0})(\varepsilon -1)-\Delta R_{2\text{E}}^{*}TE-V_{0}\varepsilon \Delta R_{2\text{I}}^{*}TE]$$

Dividing by *S*_0_ (as defined in Eq. (B1)), we obtain

(B7) $$\frac{\Delta S}{S_{0}}=\frac{S_{\text{E}}}{(1-V_{0})S_{\text{E}}+V_{0}S_{\text{I}}}[(V_{0}-V)(1-\varepsilon )-\Delta R_{2\text{E}}^{*}TE-V_{0}\varepsilon \Delta R_{2\text{I}}^{*}TE]$$

$$=\frac{1}{1-V_{0}+V_{0}\varepsilon }[(V_{0}-V)(1-\varepsilon )-\Delta R_{2\text{E}}^{*}TE-V_{0}\varepsilon \Delta R_{2\text{I}}^{*}TE]$$

For small *V*_0_, the initial factor is close to one and can therefore be eliminated. This gives us an expression for BOLD signal change that is very similar to Eq. (B5) in Obata et al. (2004) except that the volume factor differs in the intravascular term:

(B8) $$\frac{\Delta S}{S_{0}}\approx -\Delta R_{2\text{E}}^{*}TE-V_{0}\varepsilon \Delta R_{2\text{I}}^{*}TE+(V_{0}-V)(1-\varepsilon )$$

We now use the approximations Δ*R*_2E_* = 4.3*ϑ*_0_ *V*_0_ *E*_0_(*q* − *1*) and $\Delta R_{2\text{I}}^{*}=r_{0}E_{0}\left(\frac{q}{v}-1\right)$ provided by Obata et al. (2004) in their Eqs. (A8) and (A11). After substitution of these approximations into Eq. (B8), we obtain our final equation:

(B9) $$\frac{\Delta S}{S_{0}}\approx V_{0}\left[k_{1}(1-q)+k_{2}\left(1-\frac{q}{v}\right)+k_{3}(1-v)\right]$$

$$k_{1}=4.3\vartheta_{0}E_{0}TE$$

$$k_{2}=\varepsilon r_{0}E_{0}TE$$

$$k_{3}=1-\varepsilon$$

This equation retains the functional non-linear form of the classical model but uses expressions for the coefficients as provided in Obata et al. (2004).

## Footnotes

1 Clearly the concept of models of distributed neural responses that include both neural state equations and forward equations is not restricted to any particular measurement modality. Indeed, DCM implementations other than for fMRI have been developed, e.g. for event-related potentials measured with EEG/MEG (David et al. 2006) or invasively measured local field potentials (LFPs; Moran et al. 2007).

2 Using BMS, we directly compared BMs with the corrected coefficients in Eq. (3) against the original coefficients in Eq. (2). Model comparison showed that the correction in Eq. (3) yields a substantial improvement (group Bayes factor > 10^11^). The original coefficients from Eq. (2) are therefore not further considered in this paper.

3 Subsequent work by Mandeville et al. (1999) indicated that this approximation may not be perfect for transient BOLD responses, and Buxton et al. (2004) offered a more complex formulation of viscoelastic effects on blood outflow. In this study, we retained the original approximation by Grubb et al. (1974). This is supported by recent results based on BMS (Jacobsen et al., in press) which showed that the more complex model by Buxton et al. (2004) showed inferior generalizability, compared to the simpler model by Grubb et al. (1974).

4 Model comparison based on the evidence is appropriate if all models have identical *a priori* probabilities. If this is the case, as in the present study, the model evidence *p*(*y*|*m*) is identical to the posterior probability of the model, *p*(*m*|*y*).

5 This is also true for first-level Bayesian tests since these take into account the conditional dependencies among the parameters (cf., Friston et al. 2003).
